# Impact of soil legacy on plant–soil feedback in grasses and legumes through beneficial and pathogenic microbiota accumulation

**DOI:** 10.3389/fmicb.2024.1454617

**Published:** 2024-11-11

**Authors:** Mohamed Idbella, Ahmed M. Abd-ElGawad, Fatima Ezzahra Chouyia, Giuliano Bonanomi

**Affiliations:** ^1^College of Agriculture and Environmental Sciences, AgroBioSciences (AgBS) Program, Mohammed VI Polytechnic University, Ben Guerir, Morocco; ^2^Plant Production Department, College of Food & Agriculture Sciences, King Saud University, Riyadh, Saudi Arabia; ^3^Department of Agricultural Sciences, University of Naples Federico II, Naples, Italy; ^4^Task Force on Microbiome Studies, University of Naples Federico II, Naples, Italy

**Keywords:** plant–soil feedback, plant functional groups, grasses, legumes, soil chemistry, soilborne pathogens, rhizobia

## Abstract

Plants shape their surrounding soil, influencing subsequent plant growth in a phenomenon known as plant–soil feedback (PSF). This feedback is driven by chemical and microbial legacies. Here, we cultivated six crops from two functional groups, i.e., three grasses (*Lolium*, *Triticum*, and *Zea*) and three legumes (*Glycine*, *Lens*, and *Medicago*), to condition a living soil. Subsequently, the same species were sown as response plants on conspecific and heterospecific soils. We employed high-throughput sequencing in tandem with soil chemistry, including total organic matter, pH, total nitrogen, electrical conductivity, phosphorus, and macro and micro-nutrients. Our results showed that *Glycine* exhibited the strongest negative PSF, followed by *Triticum* and *Zea*, while *Lolium* displayed low feedback. Conversely, Lens demonstrated robust positive PSF, with *Medicago* exhibiting slight positive feedback. Soil chemistry significance indicated only higher Cl content in *Triticum* soil, while *Lens* displayed higher Zn and Mn contents. Microbial diversity exhibited no significant variations among the six soils. Although conditioning influenced the abundance of functionally important microbial phyla associated with each plant, no specificity was observed between the two functional groups. Moreover, each crop conditioned its soil with a substantial proportion of fungal pathogens. However, co-occurrence analysis revealed a strong negative correlation between all crop’s biomass and fungal pathogens, except *Glycine*, which exhibited a strong negative correlation with mutualists such as *Arthrobacter* and *Bacillus*. This underscores the complexity of predicting PSFs, emphasizing the need for a comprehensive understanding of plant interactions with both pathogens and mutualists, rather than focusing solely on host-specific pathogens.

## 1 Introduction

Plants can differentially influence their soil environment by altering its physical, chemical, and biological features, thereby affecting their performance relative to competitors and ultimately leading to changes in plant community composition and diversity (Aqeel et al., 2023) through a belowground process called plant–soil feedback (PSF). A particular plant species may modify its soil environment in a way that enhances its growth rate compared to other plant species, resulting in positive PSF, or in a manner that diminishes its growth rate relative to that of other plant species, resulting in negative PSF (Van der Putten et al., 2013). Positive PSF may arise from improved nutrient availability (Chapman et al., 2005) or the accumulation of symbiotic mutualists in the rhizosphere (Idbella et al., 2021). Negative PSF may be attributed to the immobilization or depletion of nutrients (Zhang et al., 2023), the build-up of root herbivores and soilborne pathogens (Van der Putten, 2003), or the accumulation of autotoxic factors (Idbella et al., 2024). PSF describes the net effect of these concurrent events, i.e., positive and negative effects, as they do not occur in isolation (Harrison and Bardgett, 2010). Variations in the strength of PSFs between species can predict the distribution of species abundance, with rarer species generally having more intense negative PSF (Bennett et al., 2017).

Among the mechanisms that cause PSF, the two most cited are plant-mediated nutrient cycling (e.g., abiotic factors) and plant-microbial interactions (e.g., biotic factors). Plants exert different effects on local nutrient cycling, and studies often suggest that litter decomposability is an important plant trait controlling plant-mediated nutrient cycling (Bonanomi et al., 2023). In natural ecosystems, litter may leave physical, chemical, and biotic legacies in the soil that strongly impact soil functioning and plant growth (Ehrenfeld et al., 2005; Bonanomi et al., 2021). In particular, the production of nutrient-rich, lignin-poor litter that decomposes rapidly creates positive PSF by promoting rapid nutrient cycling, especially when the benefits act more strongly on the plant itself (Lehmann and Kleber, 2015). On the other hand, direct interactions between plants and soil microbes show that plants differ in their local microbial communities and their response to specific microbial species (Chouyia et al., 2022). The main categories of soil microbiota that characterize PSF are natural enemies (i.e., soil microbial pathogens and pests), symbionts (i.e., mycorrhizal fungi, endophytes, nitrogen-fixing, and plant growth-promoting microbes), and decomposers (i.e., microbiota that degrade litter, root exudates, and soil organic matter) (Wardle, 2002). They can all influence plant growth directly and indirectly through their influence on soil physicochemical properties (Idbella et al., 2022b). A positive PSF can occur when the plant promotes the population growth of symbionts in different ways compared to enemies during cultivation (Klironomos, 2002) or when the promoted enemies have stronger effects on competitors than on the plant itself (Bever et al., 2010). Negative PSF occurs when the plant differentially suppresses the population growth of symbionts compared to enemies, or when facilitated symbionts have stronger effects on competitors than on the plant itself. Abiotic PSF effects are likely to be less species-specific (Heinze et al., 2020), whereas biotic PSF effects are supposed to be highly specific (Van der Putten, 2003).

Another crucial factor cited in the literature to explain the increase in negative PSF is the release of autotoxic compounds during the decomposition of plant litter (Cesarano et al., 2017). By definition, autotoxicity causes negative PSF by inhibiting the growth of conspecifics. In some cases, autotoxic chemicals also inhibit mutualistic microbes and neutralize positive PSF (Zhou et al., 2018). However, two main criticisms of the autotoxicity hypothesis have been raised. The first states that toxins from plant residues are rapidly degraded by microbial activity in the soil and become ineffective after a few weeks, while negative PSF may persist in the field for months or even years. The second states that many, if not all, organic compounds extracted from diseased soils and plant residues exhibit general phytotoxicity, which contrasts with the species-specificity of negative PSF. Alternatively, Mazzoleni et al. (2015) reported that fragmented extracellular self-DNA accumulated in litter and soil during the decomposition of conspecific residues has species-specific inhibitory effects on various wild plants. These results provide a chemical basis for autotoxicity that must be considered in explaining the negative PSF.

In agroecosystems, ecological resilience and resistance can be enhanced by improving system diversity through practices such as crop rotation, intercropping, cover crops, or the integration of livestock (Liebman and Schulte, 2015; Murrell, 2017). It has long been known that PSFs influence agricultural production and form the basis for crop rotation (Van der Putten et al., 2013). Negative PSFs, resulting from the accumulation of plant pathogens, often lead to yield decline in continuous monoculture farming. Crop diversification, including intercrops and rotations, reduces the incidence of soil pathogens by disrupting their biological cycle (Letourneau et al., 2011). This practice also improves soil microbial biomass and functions, including beneficial microbiota like arbuscular mycorrhizal fungi (AMF) (Lacombe et al., 2009) and nitrogen fixers (Li et al., 2016). Moreover, crop rotation induces changes in nutrient cycling processes (McDaniel et al., 2014), which can have variable indirect effects on pathogens and mycorrhizal fungi. For instance, increased nutrient availability might stimulate pathogen growth due to heightened host plant productivity and tissue quality (Nordin et al., 2006). However, these effects might suppress mycorrhizal fungi due to shifts in the nutrient limitation status of the microbes (Treseder and Allen, 2002; Johnson, 2010). Mechanisms for this influence involve variation in litter chemistry, soil pH, and nutrient contents among crops (Fierer and Jackson, 2006). Recent research has shown that the direction and magnitude of PSFs are influenced by the agricultural management system (Johnson et al., 2017) and the phylogenetic distance between interacting species (Miller and Menalled, 2015). In this context, legume-grass mixtures are considered an essential element of crop rotation in many agricultural systems in temperate and tropical environments, especially for organically managed farms (Holcombe et al., 2020). While grass monocultures have often been preferred by producers due to easier weed and grazing control (Beuselinck et al., 1994), the cost of nitrogen fertilizer and potential negative environmental impacts of nitrogen application have led to an urgent need to maintain or increase pasture production while reducing nitrogen fertilizer use (Solomon et al., 2011). This has resulted in increased interest in grass-legume mixed pastures. Legumes, as nitrogen fixers, can increase nutrient availability to other plants, producing interspecific positive PSF effects (Harrison and Bardgett, 2010). Similarly, grasses with highly branched roots may provide a more suitable habitat for root-associated microbes that have positive effects on other plants (Latz et al., 2015). However, an increase in root surface area, common in grasses, could also lead to an increase in the abundance of plant antagonists such as root pathogens. Still, root pathogens of grasses are specialized in monocots and are unlikely to negatively affect plants from any other functional group (Cortois et al., 2016). Nevertheless, the presence of grass endophyte symbioses can affect legume establishment (Stevens and Hickey, 1990). For example, negative PSF effects have been reported in *Medicago sativa* L., *Trifolium pratense* L., *Lotus tenuis* L., and *Trifolium repens* L. when grown with endophyte-infected tall fescue (Hoveland et al., 1999; Liu et al., 2021). These studies suggest that these negative effects are caused by the presence of endophytes and their influence on the competitiveness of the host grass, altering several host traits that may affect legume success and their interaction with rhizobia and AMF (García-Parisi et al., 2017; Idbella et al., 2019, 2021). Therefore, it is important to investigate the mechanisms by which grasses can inhibit legume establishment.

Most studies examining PSF effects in agroecosystems have focused on the performance of natural species in soils conditioned by conspecifics and heterospecifics (Klironomos, 2002; Seipel et al., 2019; Idbella et al., 2022a). Consequently, there is limited knowledge about how soil conditioning impacts plant performance in agroecosystems (Ehrenfeld et al., 2005; Bezemer et al., 2006). In this study, we investigated how individual plant species promoted or inhibited conspecific and heterospecific growth through changes in the soil. Specifically, we cultivated six plant species, including three grasses (*Triticum durum* L., *Zea mays* L., and *Lolium perenne* L.) and three legumes (*Medicago sativa* L., *Glycine max* L., and *Lens culinaris* L.). The pots were exposed to conditioning for 1 year. Following the conditioning phase, all plant communities were removed from the soil, and the same species were sown as response plants in combinations allowing the growth of each plant species on conditioned conspecific and heterospecific soils. While most studies assessing feedback effects use soil sterilization or the addition of soil inoculum to sterilized background soil, our approach involved a “whole feedback” approach. This method, based on conditioning the soil, mimics real field conditions where changes in both microbiota and soil chemistry occur during conditioning. Thus, both alterations in soil biota and chemistry contribute to PSF. To evaluate the relative importance of soil chemistry and microbiota, we fully characterized soil chemical properties, as well as fungal and bacterial communities, using next-generation sequencing. The study aimed to test the effects of different soil legacies established during the conditioning phase by each plant species on the chemical and microbial properties of the soil. Consequently, we sought to understand the impact on the growth of conspecifics and heterospecifics during the response phase. Our hypothesis posited that each species would experience species-specific negative PSF, while legumes would induce positive PSF on grasses and vice versa. Specific aims were:

- to provide evidence that in the response phase, grasses and legumes would grow less in soils that were dominated by their own functional type;
- to assess if the nature of negative PSF is associated with soil pathogens accumulation;
- to assess if positive PSF is caused by increased soil N and other major nutrients.

## 2 Materials and methods

**Figure 1:**
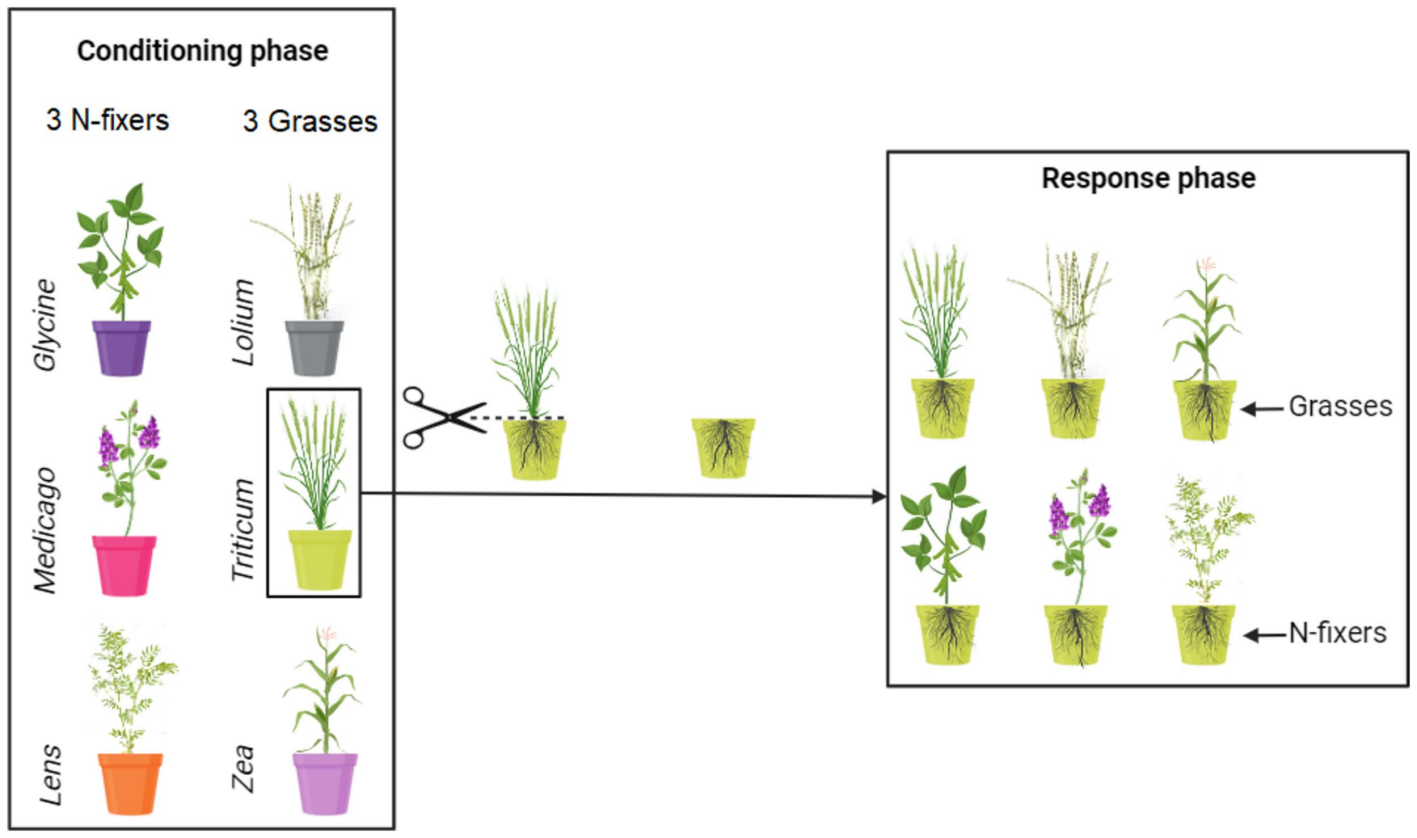
Conceptual representation of the experimental design. Soil was conditioned by monocultures of six crops. The six soil conditioning treatments are identified by having different colors of the pots. Thirty independent soil replicates were made for each of the conditioned soils. After the conditioning phase, five replicates of each of the conditioned soils were used to grow each of the six response crops, resulting thus in a combination of six response crops growing in each of the six conditioned soils. For more details, see the main text.

The experiment was carried out in a greenhouse between March 2019 and February 2021 and comprised two phases: the conditioning and the response phase (Figure 1). During the conditioning phase, soil was individually conditioned by six crop species: *Triticum durum* L. var. Desf., *Zea mays* L. var. guasconensis, *Lolium perenne* L. var. aristatum, *Medicago sativa* L. var. vulgaris, *Glycine max* L. var. Merr, and *Lens culinaris* L. var. Medik. In the response phase, the six crop species were cultivated in soil previously conditioned by the same crop, i.e., conspecific, or by each of the other five crop species, i.e., heterospecifics, for an entire growth cycle. The seeds utilized in this experiment were obtained from commercially purchased seeds with no prior treatment (De Corato sementi^R^).

### 2.1 Conditioning phase

In this initial experimental phase, plants are cultivated in the soil for a specific duration, corresponding to approximately 6 months, to condition it and modify local biotic and abiotic soil properties (Ehrenfeld et al., 2005; Van der Putten et al., 2013). Pots (25 L = 38.5 cm opening diameter × 35 cm height × 30 cm bottom diameter) were filled with sterile soil characterized by the following properties: 22.1% clay, 56.6% silt, 21.3% sand, pH 7.74, electrical conductivity 0.32 dS m^−1^, organic carbon 15.4 g kg^−1^, total nitrogen 1.6 g kg^−1^, C/N ratio 9.6, CaCO_3_ 7.16 g kg^−1^, and available phosphorus 239 mg kg^−1^. The soil was collected from the topsoil (0–30 cm) of a farm in the Campania region, southern Italy (E: 14° 18′, N: 40° 51′; 4 m a.s.l.), homogenized, and sieved through a 1 cm mesh size to eliminate coarse fragments. Prior to the experiment, the soil underwent sterilization by autoclaving at one atmosphere pressure and 120°C for 1 h, repeated three times at 24-h intervals.

In total, the conditioning phase comprised 180 pots, organized into monocultures of 6 plant species, each with 30 replicates. Fifteen seeds were sown in each conditioning pot for every plant species. The seeds underwent surface sterilization in a 3% sodium hypochlorite solution for 1 min and were rinsed multiple times with sterile water before use (Oyebanji et al., 2009). Following germination, the number of seedlings in each pot was reduced to five, and the pots received regular watering. All pots were placed randomly in a greenhouse with 70% relative humidity, 11 h of daylight with an annual average day temperature of 18°C, and 13 h of night with an annual average night temperature of 12°C. Following each plant’s growth cycle, namely 130 days for *T. durum*, 119 days for *Z. mays*, 60 days for *L. perenne*, 150 days for *M. sativa*, 130 days for *G. max*, and 110 days for *L. culinaris*, the plants were meticulously removed from their respective pots, leaving the roots intact within the soil to maintain the integrity of the rhizosphere, which harbors a substantial portion of the microbial community. At this point, soil samples were collected from each block and then divided into two fractions: one fraction was stored at 4°C for studying the chemical properties of the soil, while the other fraction was stored at −20°C for molecular analysis.

### 2.2 Response phase

In this second experimental phase, each soil conditioned by plant species, comprising a block of 30 pots, was subdivided into 6 sub-blocks, each containing 5 replicates (Figure 1). Fifteen seeds were sown in each response pot, utilizing the same seed treatments as employed in the conditioning phase. Consequently, in this response phase, plants were cultivated in soils previously conditioned by the same plants, as well as by five heterospecifics. A total of 180 conditioned pots were utilized (6 response plants × 5 replicates × 6 conditioned blocks). Following germination, the number of seedlings in each pot was reduced to five, and the pots received regular watering. Seven months later, all the plants were harvested. The plants were cut at soil level, and the shoots were dried at 70°C for 72 h, with their dry weight subsequently recorded. It is important to note that root biomass was not measured in this study. Although root biomass is recognized as crucial in this context, the experimental design involved leaving the roots undisturbed from the conditioning phase to the response phase to preserve the rhizosphere. As a result, we refrained from measuring root biomass, as it would have been challenging to differentiate between roots associated with the conditioning and response phases.

### 2.3 Soil chemistry

After the conditioning phase, soil samples were dried in a ventilated chamber at room temperature until a constant weight was achieved. The soil underwent analysis for 15 parameters, including total organic matter (OM), pH, total nitrogen (N), and macro and micronutrients crucial for plant growth. Specifically, the following parameters were assessed: Soil electrical conductivity (EC) and pH were determined in soil-water suspensions at ratios of 1:5 and 1:2.5, respectively, using a conductivity meter and a pH meter (Czekała et al., 2016). Total N was determined using the Kjeldhal method (Czekała et al., 2016), while phosphorus was determined using the molybdovanadate-phosphate method (AOAC, 1990). OM content was determined by weight loss at 550°C for 8 h (Silva et al., 2014). Potassium (K), magnesium (Mg), iron (Fe), manganese (Mn), calcium (Ca), sodium (Na), copper (Cu), and zinc (Zn) were determined by flame atomic absorption spectroscopy (Peters et al., 2003). Total limestone (CaCO_3_) was determined by the weight method against a strong acid. Attack of the limestone resulted in the release of CO_2_, the volume of which was measured (LANO: NF ISO 10693). Finally, the chloride content (Cl) in the soil was determined by the volumetric method as described by Meldrum and Forbes (1928).

### 2.4 Microbial DNA extraction and amplicon sequencing

The microbiome of three soil replicates for each plant species post the conditioning phase underwent analysis via Illumina high-throughput sequencing. Microbial DNA was extracted from 0.5 g of homogenized soil using the DNeasy PowerSoil kit (Qiagen). High-throughput sequencing of the amplified V3-V4 regions of the 16S rRNA gene (~460 bp) and ITS1-2 (~300 bp) were used to assess bacterial and fungal diversity. PCR was conducted with primers S-D-Bact-0341-b-S-17/S-D-Bact0785-a-A-21 (Berni Canani et al., 2017) and BITS1fw/B58S3-ITS2rev (Bokulich and Mills, 2013) under conditions detailed in the original studies. For bacterial primers S-D-Bact-0341-b-S-17 (5′-CCTACG GGNGGCWGCAG-3′) and S-D-Bact-0785-a-A-21 (5′-GAC TACHVGGGTATCTAATCC-3′), PCR conditions comprised 25 cycles of 95°C for 3 min, 95°C for 30 s, 55°C for 30 s, 72°C for 30 s, 72°C for 5 min, and then held at 4°C. Regarding fungal primers BITS1fw (5’-ACCTGCGGARGGATCA-3′) and B58S3-ITS2rev (5’-GAGATCCRTTGYTRAAAGTT-3′), PCR conditions included 35 cycles of 95°C for 30 s, 55°C for 30 s, and 72°C for 60 s, with a final extension of 72°C for 5 min. PCR products underwent purification using Agencourt AMPure beads (Beckman Coulter, Milan, IT) and quantification via an AF2200 Plate Reader (Eppendorf, Milan, IT). Equimolar pools were created, and sequencing was performed on an Illumina MiSeq platform, generating 2× 250 bp paired end reads.

Bacterial and fungal sequences were analysed using the DADA2 package (version 1.16.0 pipeline) (Callahan et al., 2016) in R software (4.0.4) (R Core Team, 2016). DADA2, known for its superior taxonomic resolution, retains unique sequences and calculates sequencing error rates instead of clustering to 97% similarity (Hugerth and Andersson, 2017). The resulting taxonomic units are referred to as amplicon sequence variants (ASVs) rather than operational taxonomic units (OTUs). For bacterial sequences, both forward and reverse reads were trimmed to 240 bp, and primer sequences were removed. The following filter parameters were applied: maxN = 0, maxEE for both reads = 2, truncQ = 2 (MaxEE corresponds to the maximum expected errors, TruncQ represents the parameter truncating reads at the first occurrence of a quality score less than or equal to two, and MaxN is the maximum number of ‘N’ bases accepted). Error rates were estimated with learnErrors using nearly 4 million reads. Sequences were dereplicated using derepFastq with default parameters, and exact sequence variants were resolved using the dada algorithm. The RemoveBimeraDenovo function was then employed to eliminate chimeric sequences. For fungal sequences, the pipeline included a preliminary step of trimming adapter sequences and low-quality ends (<Q20) using Cutadapt software (Martin, 2011). In both the bacterial and fungal datasets, reads with more than three errors in the forward reads and five errors in the reverse reads were removed. Taxonomy was assigned using assignTaxonomy based on the SILVA (v132) and UNITE (v7) databases for bacterial and fungal communities, respectively (Quast et al., 2013; Nilsson et al., 2019). *Chloroplast* and *Streptophyta* contaminants, as well as singleton ASVs, were removed, and the relative abundances of the remaining taxa were recalculated.

### 2.5 Statistical analysis and data visualization

The statistical significance of the biomass data obtained from the experiment was assessed using a two-way analysis of variance (ANOVA) to determine the main and interactive effects of the fixed factors, conditioning, and response phase, on shoot biomass. The results of the analysis of variance were further validated through the pairwise Tukey test, comparing the individual means of response plants in each soil history. Moreover, the plant–soil feedback effect was quantified as the ratio between the total biomass of the conditioned and response soil (Brinkman et al., 2010). In this experiment, the feedback effect was calculated as ln (total biomass of a response plant growing in soils conditioned by the same plant / total biomass of the same response plant growing in soils conditioned by a different plant). To examine significant changes in the feedback effect, the interaction data were analysed using a generalized linear model (GLM), incorporating conditioning and response status as fixed factors.

For the microbial data, alpha diversity metrics were computed, and heatmaps were generated using PRIMER 7 software (Primer-E Ltd., Plymouth, United Kingdom) to assess variations in community composition at the lowest taxonomic levels. The heatmaps visually represented the 50 most abundant taxa in the fungal and bacterial communities and organized the variables based on an association similarity index. Using a resemblance matrix calculated with Bray–Curtis dissimilarity, non-metric multidimensional scaling (NMDS) analyses, grounded on the abundance of microbial communities, were executed using the “meta.mds()” function of the vegan package in R. The vector fitting of environmental variables to NMDS ordination was ascertained utilizing the “envfit()” function of the vegan package, considering 15 major components of physical and chemical characteristics. The significance of compositional changes between the two microbial communities was tested through PERMANOVA (999 permutations), with the conditioning plant species serving as a fixed factor. Additionally, the ANOVA test was employed to assess the significance of variation in the alpha diversity metrics of the two microbial communities alongside the soil chemical characteristics. *Post hoc* Tukey tests were conducted to provide detailed insights into the level of significance between the samples. Furthermore, a Spearman ranking correlation test was applied to compare the shoot biomass of each of the six response species with soil chemical attributes, and a heatmap was generated using Rstudio (ComplexHeatmap package). Significance levels for differences were evaluated with *p* < 0.05. All statistical analyses were conducted using STATISTICA 13.3 software.

Furthermore, co-occurrence networks were established with bacterial and fungal communities based on individual ASVs to assess potential interactions or co-occurrence patterns between species and their impact on response plant biomass. The analyses were conducted for the communities of the six different conditioned soils sampled. To streamline the analysis and focus on the most abundant ASVs while minimizing the impact of rare ones, only the 50 most abundant ASVs were examined for both bacteria and fungi. Pairwise correlations between ASVs and biomass were calculated using Spearman’s correlation in R (Hmisc package). For statistical significance, only strong and significant correlations (Spearman’s r > 0.6 or r < −0.6 and *p* < 0.05) were considered. The network visualization was created using Gephi (version 0.9.2, Bastian et al., 2009), where each edge represents a robust and significant correlation, and each node represents an ASV along with the biomass node.

## 3 Results

### 3.1 Crop response to soil conditioning

**Figure 2:**
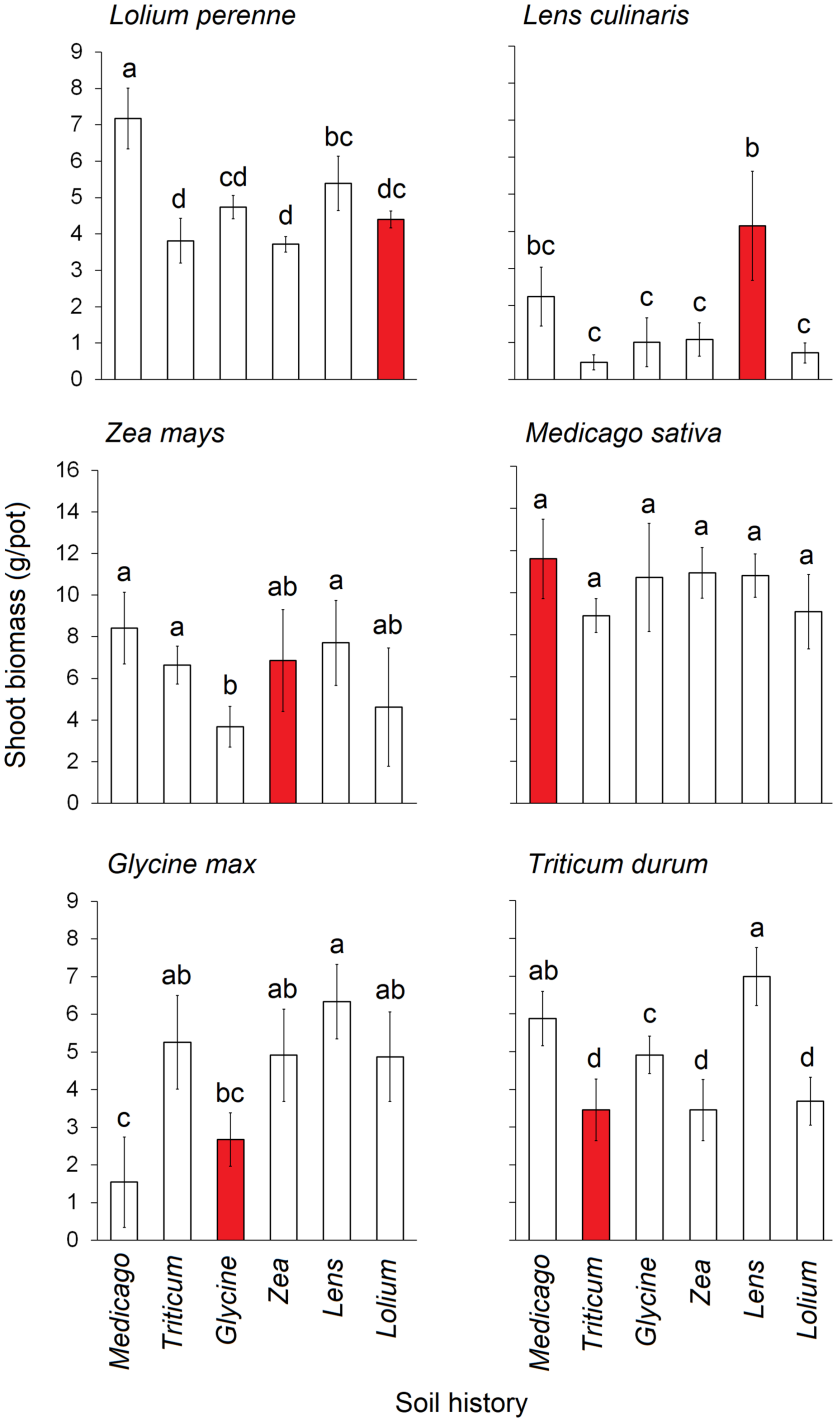
Average shoot biomass (g.pot^−1^) of each of the six response plants grown in each of the six conditioning plant species’ soil history. Red bars represent the conspecific soil history. The error bars represent the standard deviation. Bars topped by the same letter do not differ significantly by Tukey *post hoc* test (*p* < 0.05).

Shoot biomass exhibited significant differences depending on the combination of conditioning and response status (Figure 2). Specifically, the shoot biomass of *Lolium* was significantly lowest when the soil was conditioned with *Triticum*, *Zea*, and *Lolium* itself, whereas it reached its highest levels when the soil was conditioned with *Medicago*. The shoot biomass of *Lens* peaked when the soil was conditioned with *Lens* itself, while it hit its lowest levels when the soil was conditioned with *Triticum*, *Glycine*, *Zea*, and *Lolium*. *Glycine* shoot biomass was notably lower when grown in soils conditioned with *Glycine* itself and with *Medicago*. *Triticum* shoot biomass was at its lowest in soils conditioned with *Triticum*, *Zea*, and *Lolium*, and at its highest with *Lens* and *Medicago*. *Zea* shoot biomass was the lowest in *Glycine*-conditioned soil and the highest in *Medicago*, *Lens*, and *Triticum*. Notably, no significant differences were observed in the shoot biomass of *Medicago* when grown in the six conditioned soils.

### 3.2 Plant–soil feedback

**Figure 3:**
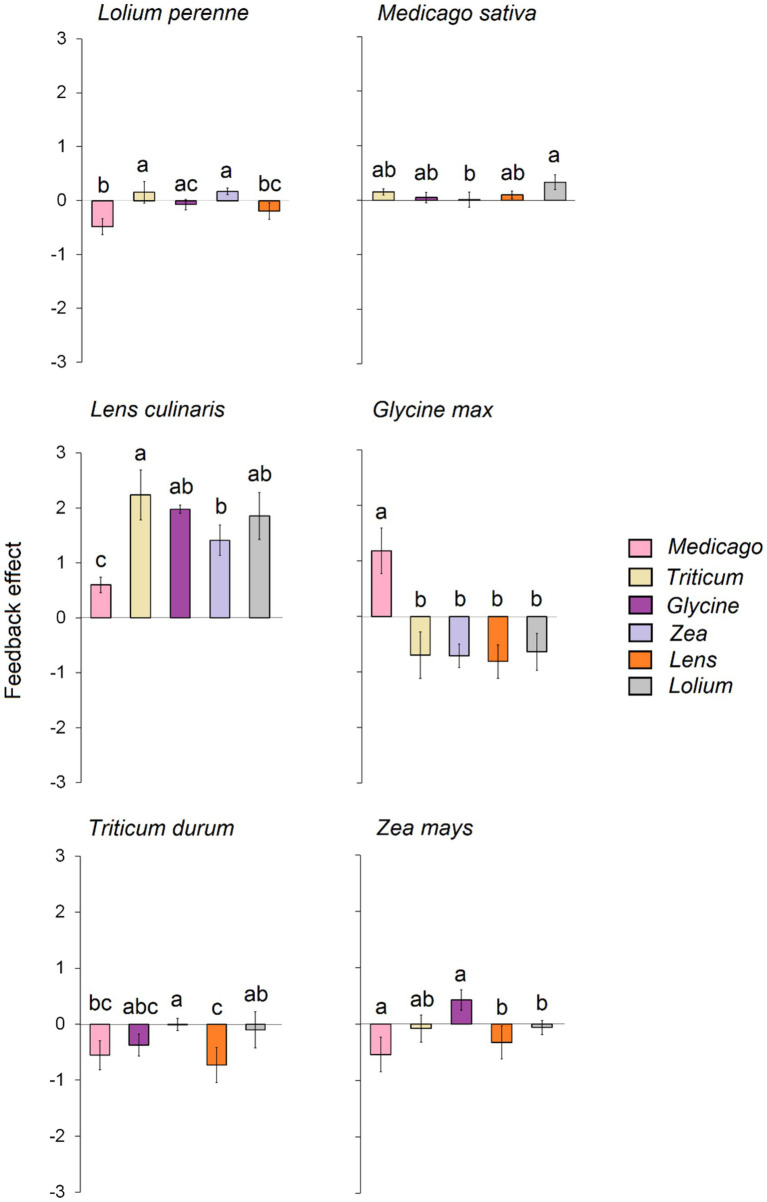
Feedback effect of each of the six response plants, i.e., biomass of each response plant grown in its own conspecific conditioned soil divided on the biomass of the same plant species grown on each of the heterospecific five conditioned soils. The error bars represent the standard deviation. Bars topped by the same letter do not differ significantly by Tukey *post hoc* test (p < 0.05).

Our results reveal that the tested plant species exhibited varying feedback depending on the conditioned soil (Figure 3). In detail, significant negative feedback was observed for *Lolium* when grown on soil conditioned with *Medicago* and *Lens*. In contrast, significant positive feedback was generated when the soil was conditioned with *Triticum* and *Zea*. Similarly, *Zea* experienced significant negative feedback when grown on soil conditioned with *Medicago*, *Lens*, and *Lolium*, while significant positive feedback occurred when grown on soil conditioned with *Glycine*. On the other hand, *Triticum* and *Glycine* displayed strong negative feedback when grown in all conditioned soils, except for the response of *Glycine* in a conditioned *Medicago* soil, which exhibited strong positive feedback. *Lens* and *Medicago* showed significant positive feedback when grown in each of the six conditioned soils. In general, the overall feedback effect indicated that *Glycine* exhibited the strongest negative feedback, followed by *Triticum*, while low feedback was recorded for *Lolium* (Supplementary Figure S1). On the other hand, *Lens* was the only crop that showed strong positive feedback, while slight positive feedback was recorded for both *Zea* and *Medicago*.

### 3.3 Soil chemical properties and correlation with crop response

Soil chemical parameters exhibited slight variations among the conditioned soils (Table 1). Specifically, *Triticum* conditioned soil demonstrated the highest Cl content, but lower OM and Mn contents compared to the other conditioned soils. Conversely, *Lens* conditioned soil displayed the highest levels of Zn and Mn compared to the other soils. No significant differences were identified between the soils in terms of the remaining chemical parameters.

**Table 1:** Soil chemical characterization of each of the six soil histories, different letters indicate statistically significantly differences by Tukey *post hoc* test (*p* < 0.05).

	Zea	Lens	Glycine	Triticum	Lolium	Medicago
pH	8.05a	8.09a	8.11a	8.27a	8.22a	8.17a
Total limestone (%)	1.67a	1.58a	2.68a	2.38a	1.20a	1.89a
EC (mS/cm)	0.22a	0.22a	0.20a	0.31a	0.23a	0.20a
Cl (g/Kg)	0.07ab	0.06b	0.06b	0.20a	0.07ab	0.06b
Na (g/Kg)	0.33a	0.30a	0.32a	0.38a	0.50a	0.34a
OM (%)	3.39ab	3.41ab	3.57ab	3.33b	3.58ab	3.68a
Total N (%)	0.19ab	0.18b	0.19ab	0.19ab	0.19ab	0.19a
P (mg/Kg)	134.32a	136.58a	135.23a	141.23a	130.91a	124.93a
K (g/kg)	2.08a	2.12a	2.06a	2.28a	2.35a	2.43a
Mg (g/Kg)	0.55a	0.56a	0.56a	0.57a	0.58a	0.56a
Ca (g/Kg)	6.34a	6.55a	6.22a	6.63a	6.37a	6.11a
Cu (mg/Kg)	40.95a	41.49a	39.40a	39.65a	39.80a	39.66a
Zn (mg/Kg)	20.94ab	24.18a	19.70ab	19.49ab	18.90b	17.76b
Mn (mg/Kg)	31.02ab	32.13a	30.46ab	26.33b	31.92ab	30.43ab
Fe (mg/Kg)	31.56a	31.65a	31.51a	43.63a	32.30a	29.84a

The Pearson correlation between shoot biomass and soil chemical parameters (Supplementary Figure S2) revealed a strongly significant positive correlation between Fe, Mn, Cu, Ca, Na, Cl, total N, and CaCO_3_ with *Glycine* biomass, accompanied by a strong negative correlation with soil pH. For *Lens*, biomass showed a positive correlation with Zn and K content and a negative correlation with CaCO_3_, while *Medicago* biomass was significantly correlated with soil P, EC, and total N content. *Lolium* biomass displayed a significant positive correlation with Mn, Ca, and K content, while it exhibited a negative correlation with total N content. The contents of Mn, Zn, and Cu were negatively correlated with the growth of *Triticum*, whereas Ca and soil pH demonstrated a positive correlation. Biomass of *Zea* was negatively correlated with P, Ca, K, Na, and soil pH, while it showed a positive correlation with soil Fe content.

### 3.4 Microbial diversity and community composition

**Figure 4:**
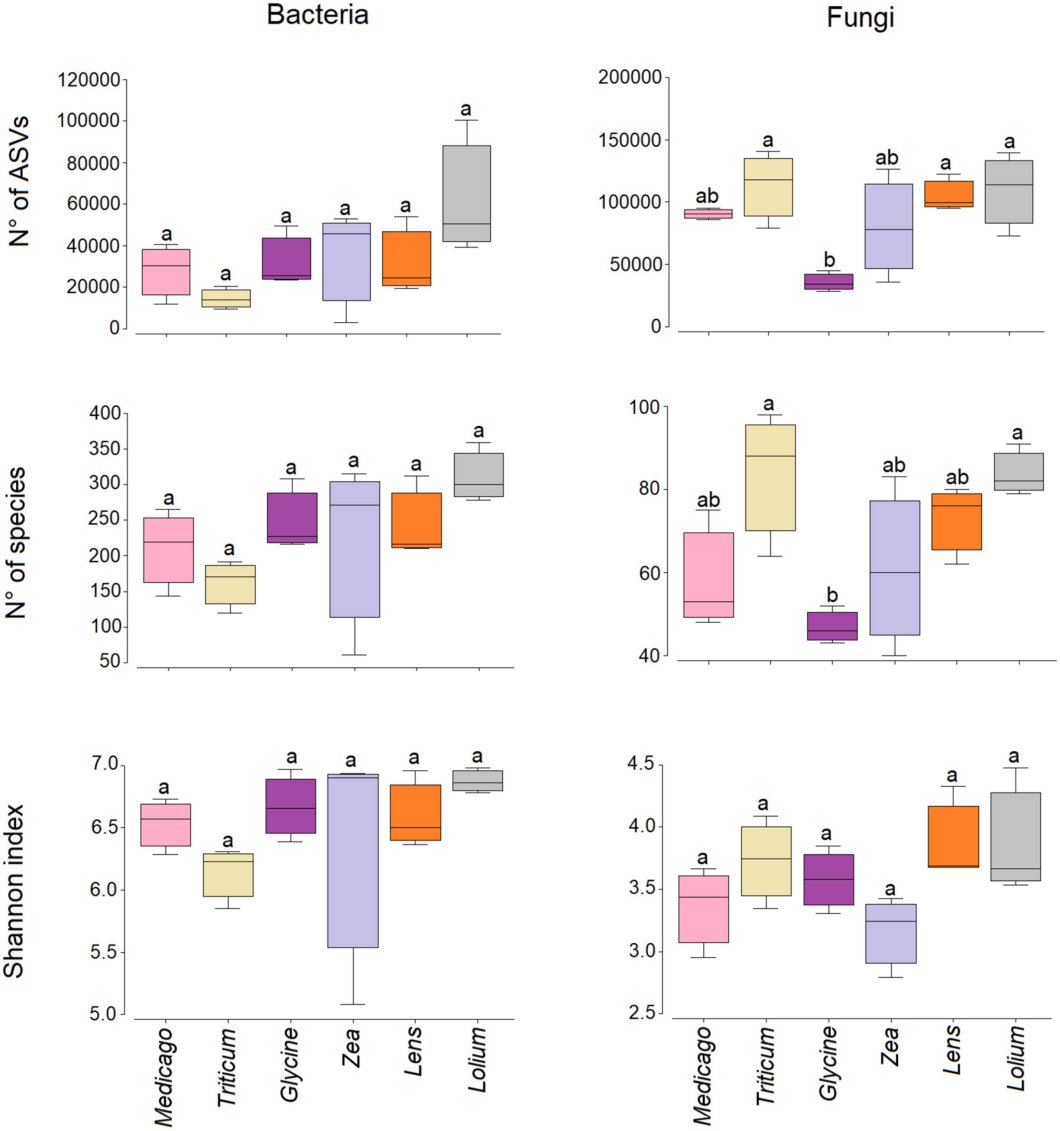
Box plots showing the variation in the Shannon diversity, number of species and reads for bacterial and fungal communities in the six soil histories. Different letters indicate significant (*p* < 0.05) differences in the indices. The lower and upper bounds of the boxplots show the first and third quartiles (the 25th and 75th percentiles); the middle line shows the median, whiskers above and below the boxplot indicate inter-quartile range.

Our results show that no significant differences were observed in the number of bacterial species, the number of ASVs and the Shannon diversity index (Figure 4). On the other hand, the number of fungal species was significantly low in *Glycine* conditioned soil compared to *Triticum* and *Lolium*, while the number of ASVs was significantly low in *Glycine*-conditioned soil compared to *Triticum*, *Lens*, and *Lolium*. In contrast, no significant change was observed in Shannon diversity index for fungi among the conditioned soils.

At the phylum level (Supplementary Figure S3), the bacterial community varied among the conditioned soils. In detail, all conditioned soils contained mainly *Pseudomonadota*, ranging from 20.6% in *Triticum* to 25.5% in *Glycine* soils. On the other hand, the highest percentage of *Actinomycetota* was found in *Triticum* soils with 18.0%, while the lowest abundance was recorded in *Glycine* soil with a percentage of 12.8%. *Planctomycetota* abundance, however, ranged from 14% in *Medicago* soil to 17.2% in *Triticum* soil. While *Gemmatimonadota* were less abundant in *Glycine* soil at 9.2%. Moreover, the highest levels of *Acidobacteriota* were found in *Glycine* soil (9.2%) followed by *Zea* soil (8.9%) while the lowest levels were found in *Lolium* (6.4%) soils. *Verrucomicrobiota*, on the other hand, was most abundant in *Glycine* soil (7.4%).

The fungal community showed a clear variation among the conditioned soils (Supplementary Figure S3). In particular, all the soils studied were dominated by the phylum *Ascomycota*, with abundance ranging from 95.1, 93.9 and 92.3% in the soils of *Triticum*, *Glycine* and *Lolium*, respectively, to 57.6% in the soils of *Zea*. However, the highest percentage of the phylum *Basidiomycota* was found in the soil of *Zea* (21.9%), while their abundance did not exceed 5% in the other soils. On the other hand, the phylum *Chytridiomycota* was found in *Lens* (10.3%) and *Zea* (10%) soils, and less than 2% in the other soils.

**Figure 5:**
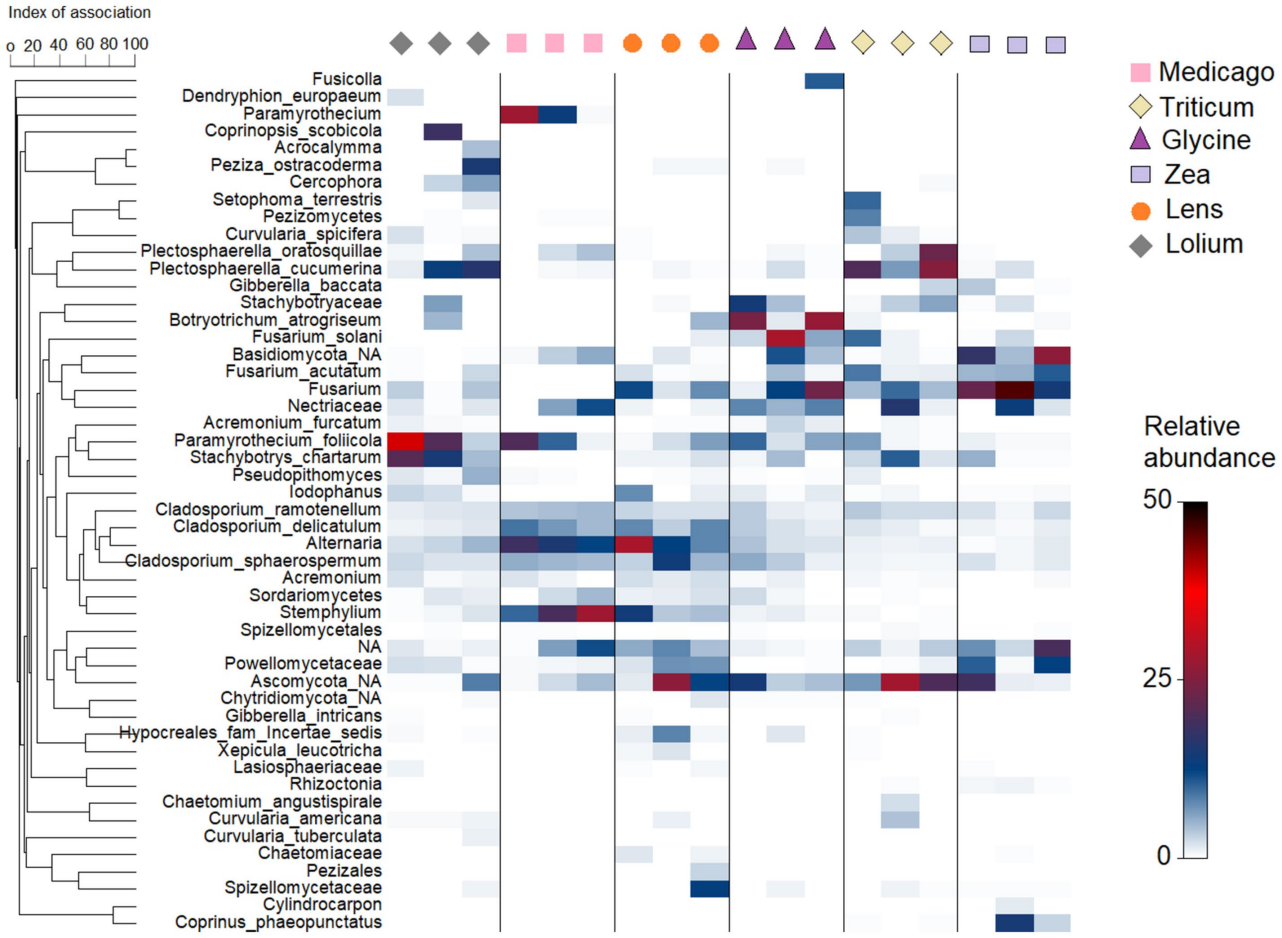
Heatmap showing relative abundance of the 50 most frequent Amplicon Sequence Variants (ASVs) in the fungal community in each of the six soil histories. The hierarchical grouping of variables is based on Whittaker’s association index.

At lower taxonomic level, the bacterial heatmap showed a slight difference between the conditioned soils with respect to the 50 most common ASVs (Supplementary Figure S4). Specifically, we found that all conditioned soils had high abundance of *Acidobacteriota* subgroup_6 and *Longimicrobiaceae*, while *Pedosphaeraceae* were more abundant in *Glycine*, *Medicago* and *Lens* than in the other soils. In addition, a large amount of *Pseudarthrobacter* was found in *Lolium*, *Zea* and *Triticum* soils, while *Bacillus* was more abundant in *Medicago* soil. On the other hand, the fungal heatmap showed a clear variation among the conditioned soils with respect to the 50 most abundant ASVs (Figure 5). In particular, *Plectosphaerella cucumerina* was more abundant in *Lolium* and *Triticum* soils, while *Plectosphaerella oratosquillae* was highly abundant in conditioned *Triticum* soil. *Botryotrichum atrogriseum*, on the other hand, was particularly abundant in *Glycine* soil. In addition, *Fusarium solani* was highly abundany in *Glycine* soil followed by *Triticum*. *Zea* soil, however, contained the highest abundance of *Fusarium acutatum*, followed by *Triticum*. Moreover, *Paramyrothecium foliicola* was highly abundant in *Lolium* and *Medicago*, while *Stachybotrys chartarum* was most abundant in *Lolium* soils. *Alternaria* and *Cladosporium delicatulum* were very common in *Medicago* and *Lens* soils. *Stemphylium* was highly abundant only in *Medicago* soil and less abundant in *Lens* soil.

### 3.5 Linking microbial community to soil chemical properties

**Figure 6:**
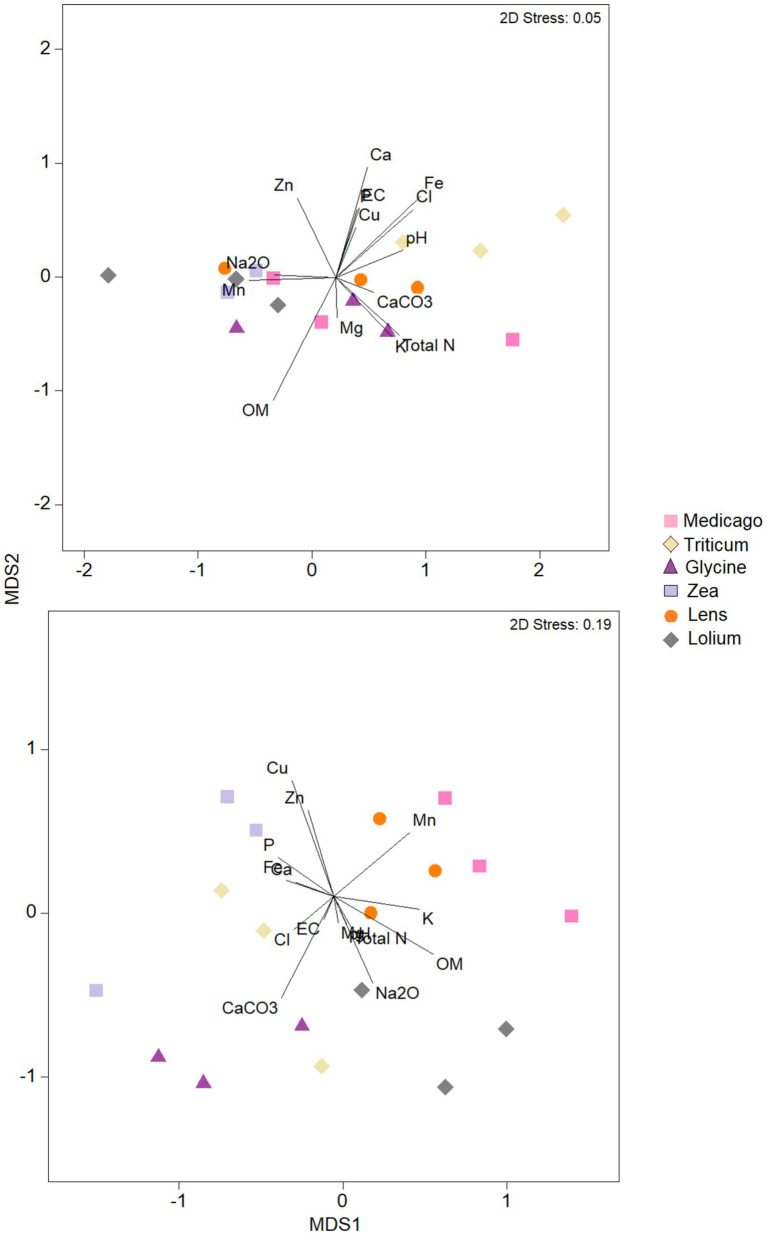
Nonmetric multidimensional scaling (NMDS) plots of bacteria and fungi communities in each of the six soil histories. MDS axis 1 and MDS axis 2 represent the two axes of the two-dimensional ordination space. Each point represents the microbiome of one replicate of the plant. The stress-level shown in each plot indicates how well the individual distances between objects are represented (between 0 and 1; the closer to 0, the better are original data points represented in the ordination space). Vectors represent soil environmental variables which significantly correlated with the ordination (*p* < 0.05 based on 999 permutations).

The nMDS analysis of the bacterial community in terms of chemical parameters (Figure 6) showed that the ordination of *Medicago*, *Glycine* and *Lens* samples was strongly correlated with soil OM content, while the ordination of *Lolium* and *Zea* samples was positively correlated with Mn, Zn, and Na content. However, pH and Fe content showed positive correlation with ordination of *Triticum* samples. The other soil chemical parameters showed negative correlation with bacterial ordination of all soil samples. As for the ordination of samples based on fungal community, nMDS analysis showed that Cu, Mn, K, pH, Na, CaCO_3_, EC, P, total N and OM were positively correlated with soil samples of *Medicago*, *Lens*, and *Lolium*. Whilst ordination based on fungal community of *Triticum*, *Glycine*, and *Zea* was positive and strongly correlated with Cl content, followed by Zn, Mg, Fe and Ca contents.

### 3.6 Linking microbial community to crop response

**Figure 7:**
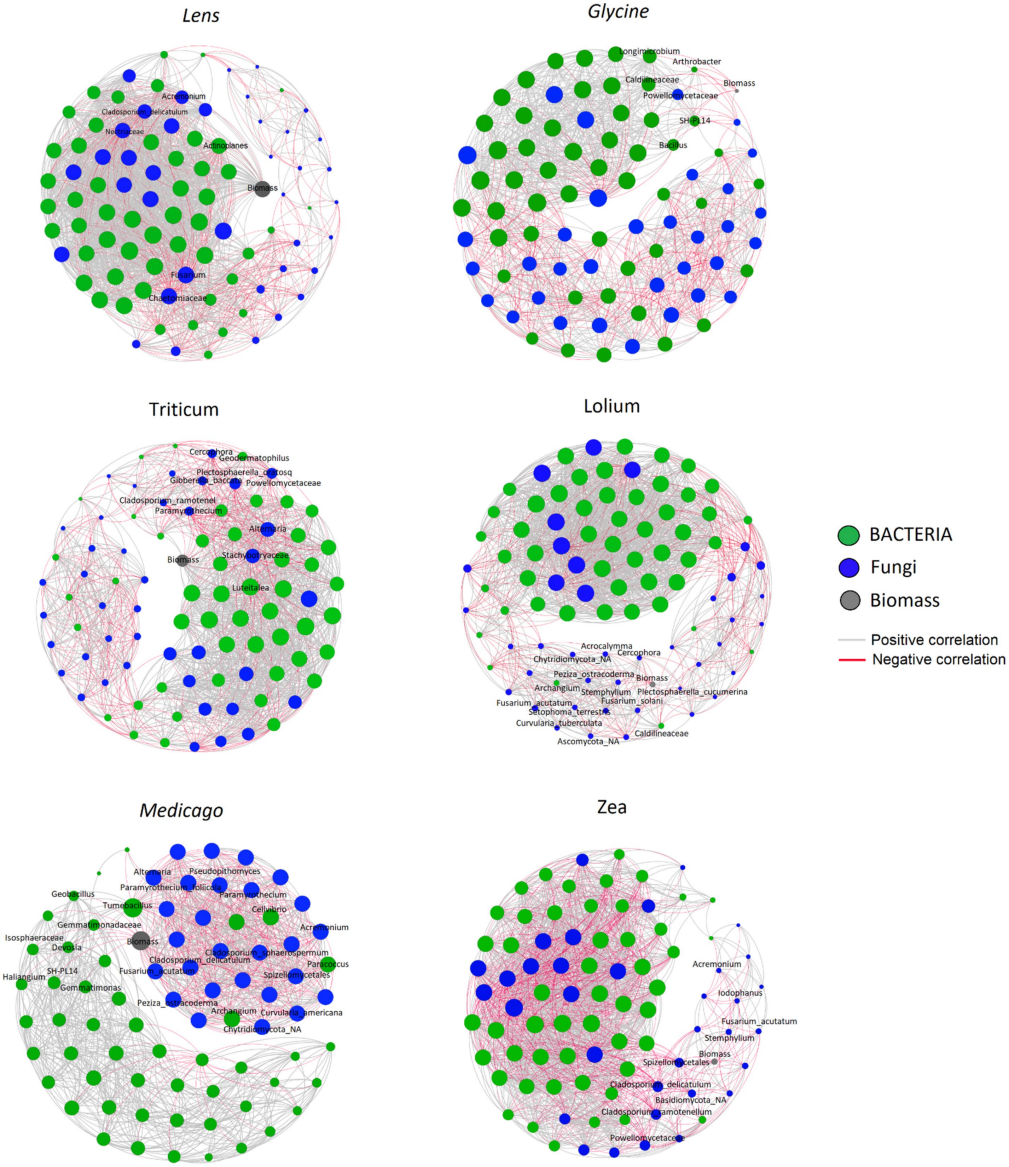
Correlation base network analysis showing potential interactions between bacterial, fungal families and response biomass in conspecific soil for each of the six response plants. The lines connecting nodes (edges) represent positive (grey) or negative (red) co-occurrence relationship. The connection stands for a strong (Spearman’s *ρ* > 0.6 and ρ < −0.6) and significant (*p*-value<0.05) correlation. The size of each node is proportional to the ASV relative abundance, only the top 50 ASVs were kept. The nodes were coloured by kingdom level.

We constructed six co-occurrence networks (Figure 7) and calculated five topological parameters to assess interactions among ASVs and with the response biomass for each of the five networks (Supplementary Table S1). The number of nodes ranged among the networks from 77 in *Medicago* to 91 in *Lolium*, whereas the number of edges ranged from 1,022 in the *Medicago* network to 1809 in *Lens*. The percentage of positive ASVs correlations in the microbial networks ranged from 57.6% in *Zea* to 78.7% in *Lolium*. The network diameter varied among soils, from the lowest value of 5 in *Lens* and *Medicago* to the highest value of 7 in *Glycine* network. Moreover, the network density was highest in *Zea* and *Lens*, while it was lowest in *Glycine*. However, the values of characteristic path length and clustering coefficient showed no significant changes among the conditioned soil networks. On the other hand, modularity recorded the highest value of 1.537 in *Zea* network while the lowest values of 0.339 and 0.452 were recorded in *Lens* and *Lolium* soils, respectively.

The co-occurrence network showed that *Glycine* biomass had significantly strong negative interactions with *Arthrobacter*, *Bacillus*, *Caldilineaceae*, *Longimicrobium*, *Powellomycetaceae* and *SH _PL14* ASVs, while *Lens* biomass was negatively correlated with *Acremonium*, *Actinoplanes*, *Chaetomiaceae*, *Cladosporium delicatulum*, *Fusarium* and *Nectriaceae* ASVs. On the other hand, *Lolium* biomass had strong negative interactions with *Acrocalymma*, *Curvularia tuberculata*, *Fusarium acutatum*, *Fusarium solani*, *Plectosphaerella cucumerina*, *Setophoma terrestris* and *Stemphylium* ASVs. *Triticum* however showed significant negative interactions with ASVs *Alternaria*, *Cladosporium ramotenellum*, *Gibberella baccata*, *Paramyrothecium* and *Plectosphaerella oratosquilla* while the ASVs that showed significant negative interactions with *Medicago* biomass are *Acremonium*, *Alternaria*, *Cellvibrio*, *Cladosporium delicatulum*, *Cladosporium sphaerospermum*, *Curvularia Americana*, *Devosia*, *Fusarium acutatum*, *Geobacillus*, *Haliangium*, *Paramyrothecium foliicola*, *Paracoccus*, and *Tumebacillus*. Finally, *Zea* biomass showed significant negative interactions with the following ASVs: *Acremonium*, *Cladosporium delicatulum*, *Cladosporium ramotenellum*, *Fusarium acutatum*, *Iodophanus*, and *Stemphylium*.

## 4 Discussion

In this study, we compared the legacies of six plant species, belonging to grass and nitrogen-fixing functional groups, on conspecific and heterospecific plant performances. We assessed soil chemical properties and soil microbiota using a whole PSF approach. Overall, we found that plant species-specific legacies affect all these variables in some way. Indeed, the response of plant species exhibited different PSF behaviors depending on the previously conditioned soil. Previous studies have shown that the direction and effect sizes of PSF appear to differ between functional plant groups (Kulmatiski et al., 2008; Meisner et al., 2014). However, the present study, contrary to our expectations, shows that PSF may not be specific to functional groups. We observed that the grass *Lolium* produces strong negative feedback when grown in soils conditioned by the legumes *Medicago* and *Lens* (i.e., lower growth of *Lolium* in soils conditioned by *Lolium* compared to the growth of *Lolium* in soils conditioned by *Medicago* and *Lens*). In contrast, *Lolium* showed strong positive feedback when grown in soils previously conditioned by the grasses *Triticum* and *Zea*. However, *Zea* grass suffered a significant negative PSF when grown in soils conditioned with the legumes *Medicago*, *Lens*, and the grass *Lolium*. A significant positive PSF occurred when grown in soil conditioned with the *Glycine* legume. Notably, *Glycine* recorded the strongest negative PSF in its own soil and negative feedback when grown in all conditioned soils except *Medicago* legume soil, which showed strong positive PSF. Existing evidence suggests that *Medicago* is particularly susceptible to autotoxicity (Chon et al., 2006), a specialized form of allelopathy. Autotoxicity involves chemical compounds from older plants affecting their own seedlings, resulting in a heightened negative PSF, which aligns with our findings. On the other hand, the grass *Triticum* suffered from a strong negative PSF in all conditioned soils without exception. Similarly, Hannula et al. (2019) concluded that the direction of the PSF could not be predicted solely from the plant group or family, even though soils from grasses tended to have more positive feedback than soils conditioned by forbs and legumes.

The observed patterns in the present study may be influenced by at least two mechanisms investigated here: soil nutrient availability and/or soil microbial communities. The advantage of using the “conspecific” and “heterospecific” feedback approach is that it can illuminate the chemical and microbial legacies produced by the decomposition of litter and root exudates of different plant species during the conditioning phase. Originally, we assumed that each plant species would alter the chemical properties of the soil in a unique way, and that these changes could affect subsequent growth. However, our results show that the differences in the chemical legacies produced by the six plant species were statistically insignificant. Legumes live in symbiosis with nitrogen-fixing rhizobacteria, and we hypothesize that the feedback effects would be due to nitrogen availability, even though we did not detect differences in nitrogen availability in the soil chemical analysis between legumes and grasses. Our results support the idea that priority effects in plant communities are not solely a matter of resource availability (Cesarano et al., 2017). Since *Triticum* and *Lolium*, which also suffered from a strong negative feedback effect, did not differ in terms of chemicals with *Lens*, which had the highest positive feedback effect, we could speculate that the diversity of conditioning plant species has little effect on subsequent plant growth by altering nutrient availability in the soil. Similarly, a recent study by Xue et al. (2021) showed that differences in soil chemical analysis between soils conditioned by four grasses, including *Lolium perenne*, and three legumes, including *Medicago Sativa* and *Trifolium repens*, had no effects on subsequent plant invasion in a PSF experiment.

Alternatively, soil microbes are thought to significantly influence PSF, both directly by affecting plant growth or defense responses, and indirectly, for example, by affecting mineralization or acting as antagonists of plant pathogens (Van der Putten et al., 2013; Chialva et al., 2018). Our results showed that microbial diversity, i.e., the Shannon diversity index, did not exhibit significant differences among the six conditioned soils. Moreover, the effect of conditioning on microbial community composition showed no specificity between the two plant functional groups. However, the abundance of functionally important microbial phyla was affected by plant legacies. We found that *Actinomycetota* were highly abundant in the soils of grass *Triticum*, while less abundant in the soils of legume *Glycine*. Moreover, *Gemmatimonadota* were less abundant in *Glycine* soils. Similarly, the highest levels of *Acidobacteriota* were found in *Glycine* soils followed by *Zea* soils, while the lowest levels were found in *Lolium* soil. *Verrucomicrobiota*, on the other hand, were most abundant in *Glycine* soils. Moreover, *Ascomycota* abundance was the highest in *Triticum* and *Glycine* soils, while the phylum *Basidiomycota* was mostly found in the soil of *Zea*. Our results indicate that each plant species, rather than plant functional groups, generates its own microbial legacy in the soil after a period of conditioning. In contrast, several recent works have shown that plant family and functional group can explain a large portion of the variation in microbial community structure (Dassen et al., 2017; Hannula et al., 2021). Moreover, Connell et al. (2021) found a significant legacy of conditioning by *Bromus inermis* that affected not only the bacterial community composition but diversity as well. We hypothesized that microbial community composition would be altered by individual plant species, possibly due to the higher amount and/or diversity of litter that falls, decomposes, and enters the soil C and N cycles (Hooper et al., 2000). Previous studies suggest that litter decomposition in the soil can alter microbial biomass, composition, and community structure by increasing substrate variability and diversity of soil chemical compounds, and that this can vary depending on the plant species (Chapman et al., 2013). These results were confirmed by our nMDS analysis showing that the ordination of the microbial community for each plant species was strongly correlated with different soil chemical parameters.

Interestingly, our results show that at the low taxonomic level, the bacterial ASVs *Pedosphaeraceae* were more abundant in the legumes *Glycine*, *Medicago*, and *Lens* soils, while *Bacillus* was more abundant in *Medicago* soils. A recent study by Yuan et al. (2022) demonstrated the significant potential of *Pedosphaeraceae* as key bacteria with interspecific interactions for phytoremediation. Additionally, *Bacillus* species, known for forming spores that can survive in soil for extended periods under harsh environmental conditions (Hashem et al., 2019), were found in high abundance in the soils of legumes due to their exclusive symbiotic ability. Moreover, our results reveal a substantial amount of *Pseudarthrobacter* in the soils of *Lolium*, *Zea*, and *Triticum* grasses. *Pseudarthrobacter* is a group of endophytic bacteria known to be present in soils, deserts, and mines (Finger et al., 2019; Chai et al., 2019). Notably, *Pseudarthrobacter sulfonivorans* strain Ar51 has demonstrated efficient degradation of petroleum and several benzene compounds at low temperatures (Zhang et al., 2016). Previous studies have emphasized that the effects of soil legacy can be explained primarily by soil fungal composition (Bezemer et al., 2006; Wang et al., 2019). However, our results indicate that the abundance of specific fungal species or groups of fungi in the soil is more crucial for plant growth and, consequently, PSF than the composition of the entire fungal community. Interestingly, we found that the soil of *Glycine* contained a high abundance of *Botryotrichum atrogriseum* and *Fusarium solani*, while the soil of *Triticum* contained a high abundance of *Plectosphaerella cucumerina*, *P. oratosquillae*, *F. solani*, and *F. acutatum*. In contrast, the soil of *Lolium* contained a high relative abundance of *P. cucumerina*, *Paramyrothecium foliicola*, and *Stachybotrys chartarum*. *Lens* soil, however, contained high levels of *Alternaria* and *Cladosporium delicatulum*, while *Medicago* soil had, in addition, high levels of *Stemphylium*. Our results indicate that each plant species conditions its own soil with a high proportion of putative pathogenic fungi, which could explain the direction of the generated PSF in the response phase. However, when linking the conspecific biomass to different ASVs present in the soil, our co-occurrence analysis showed that all plant species had a strong, significant negative correlation with fungal pathogens accumulated in the soil, with the exception of *Glycine*. *Glycine* showed a strong negative correlation with plant growth-promoting rhizobacteria such as *Arthrobacter* and *Bacillus*, suggesting that the ability to predict PSFs requires a better understanding of plant interactions with diverse communities of plant pathogens and mutualists, rather than single host-specific pathogens (Bever et al., 2012; Benítez et al., 2013).

## 5 Conclusion

In conclusion, our study on the legacies of six plant species belonging to grass and nitrogen-fixing functional groups has provided valuable insights into the interactions within soil ecosystems. We compared conspecific and heterospecific plant performances, assessed soil chemical properties, and delved into soil microbiota using a PSF approach. The observed patterns in our study suggest that the response of plant species exhibits diverse PSF behaviors depending on the previously conditioned soil. Our results highlight the complexity of plant–soil interactions and emphasize that predicting PSF only by basing on plant functional groups might be insufficient, as individual plant species play a crucial role in shaping the outcomes. Two potential mechanisms, soil nutrient availability and microbial communities, were explored to elucidate the observed patterns. While our initial assumption posited unique chemical legacies from each plant species, statistical insignificance in soil chemical differences implies complicated interactions at play. Moreover, the distinct microbial legacies generated by each plant species suggest that the microbial community composition is more influenced by individual plant species than broader functional groupings. On the other hand, soilborne pathogens were found to be abundant in all soils, and our co-occurrence analysis provides insights into the correlations between conspecific biomass and soil microbes, emphasizing the importance of understanding diverse plant-pathogen and mutualist interactions for accurate predictions of PSF. Understanding the relationships between plants and soil microbes can guide sustainable practices, fostering resilient agricultural ecosystems in the face of changing environmental conditions. Future research should investigate the roles of soilborne pathogens and mutualistic microbes across diverse ecosystems, with a focus on plant genotypes and environmental stressors, which will provide deeper insights into PSF dynamics. Moreover, long-term studies are needed to capture temporal shifts in microbial communities and soil chemistry over successive plant generations.

## Data Availability

The original contributions presented in the study are included in the article/Supplementary material, further inquiries can be directed to the corresponding author. Reads of the sequence data have been deposited in the NCBI Sequence Read Archive (SRA) with accession no. PRJNA1176467.
