# Supplementary material for: Impact of soil legacy on plant–soil feedback in grasses and legumes through beneficial and pathogenic microbiota accumulation
Source: Front Microbiol. 2024 Nov 11;15:1454617. doi: 10.3389/fmicb.2024.1454617 (PMC11586280; doi:10.3389/fmicb.2024.1454617)
Supplement: Supplementary file 1 [file Data_Sheet_1.docx]

Supplementary Material

**
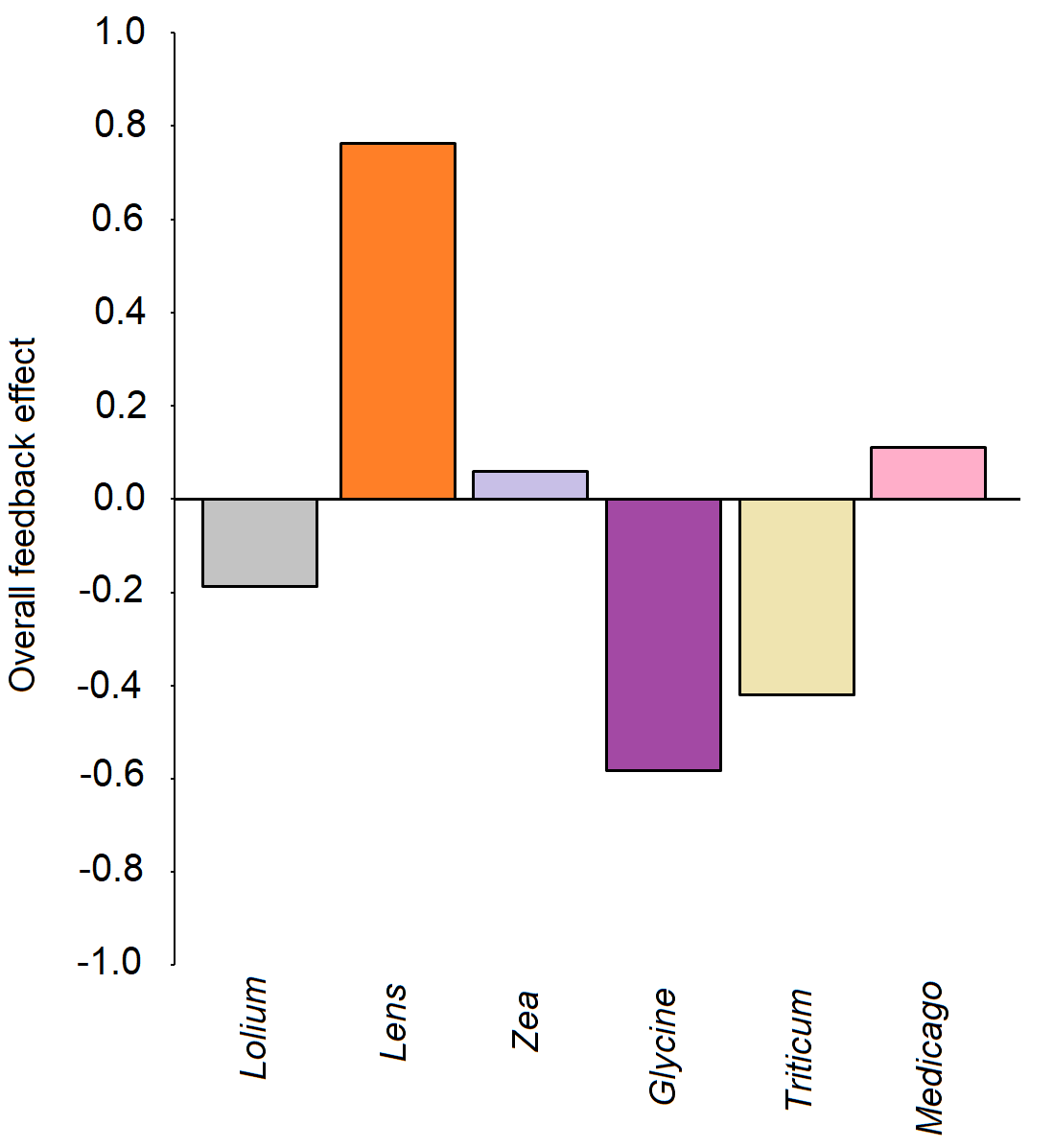
**

**Figure S1.** Overall feedback effect of the six response plant species grown in the eight conditioned soil histories.

**
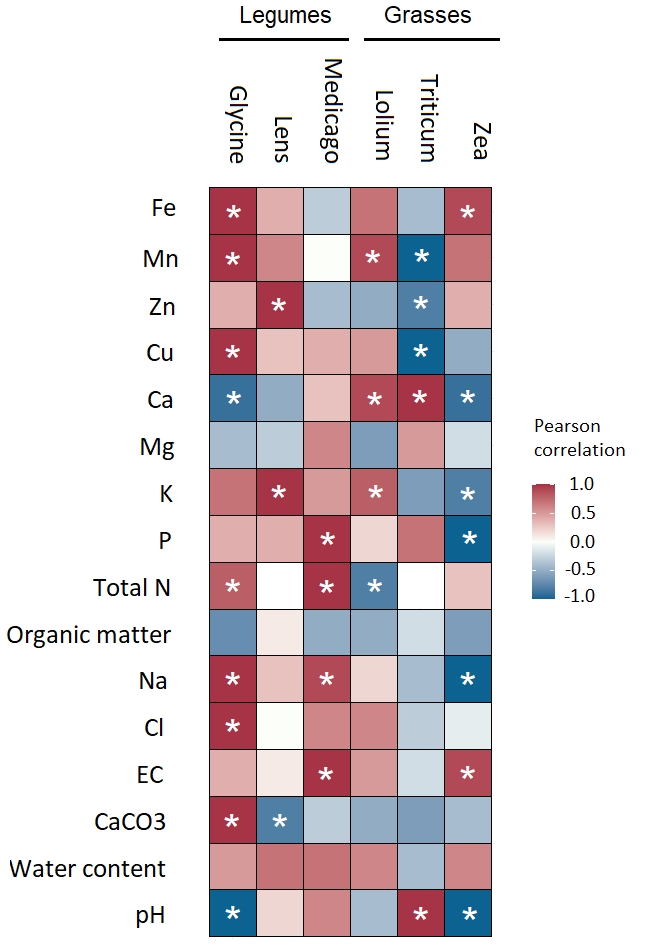
**

**Figure S2.** Pearson based correlation between conspecific crop biomass and soil chemical properties.

**
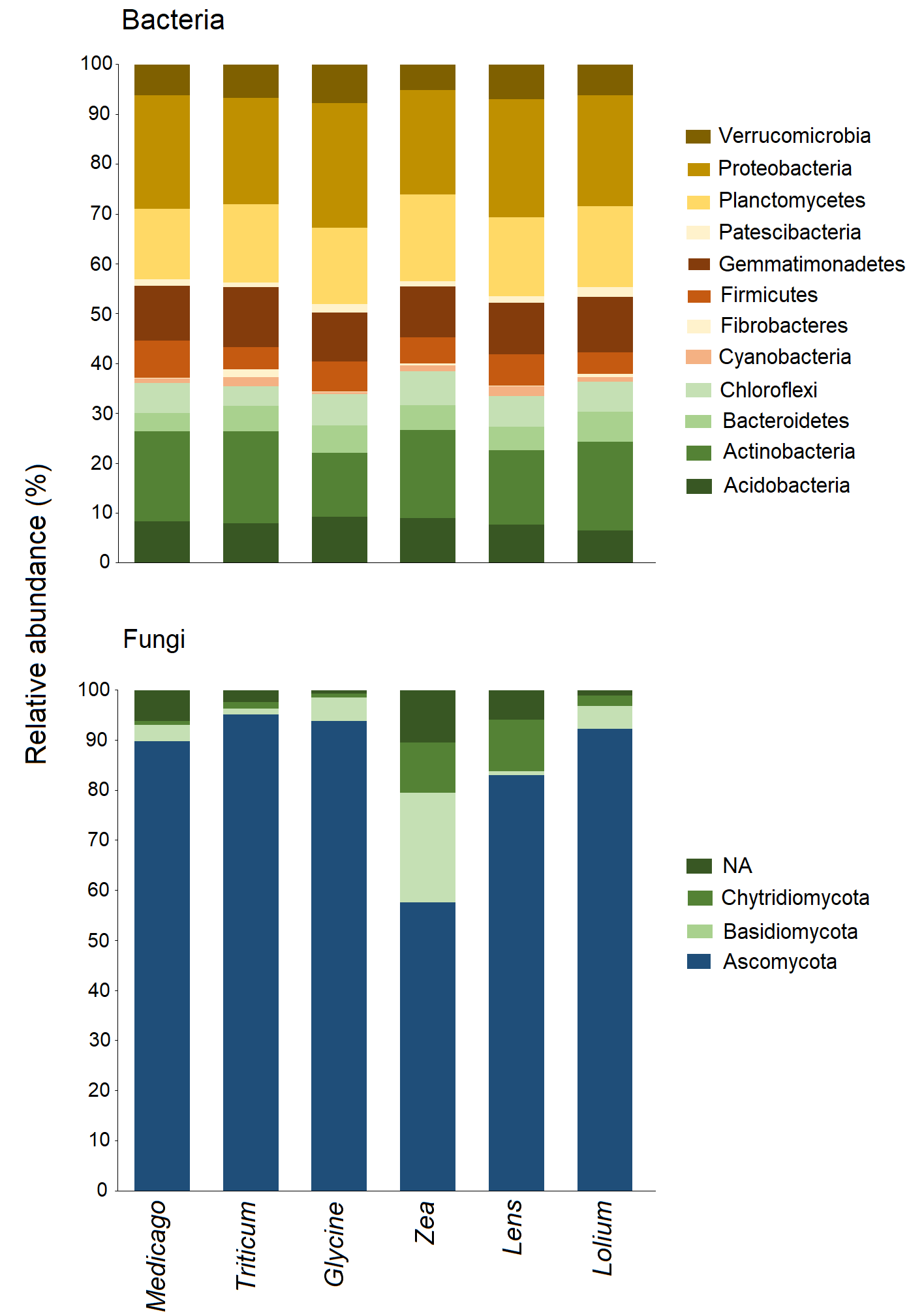
**

**Figure S3.** The relative abundance of various bacterial and fungal phyla in the soil of each soil history.

**
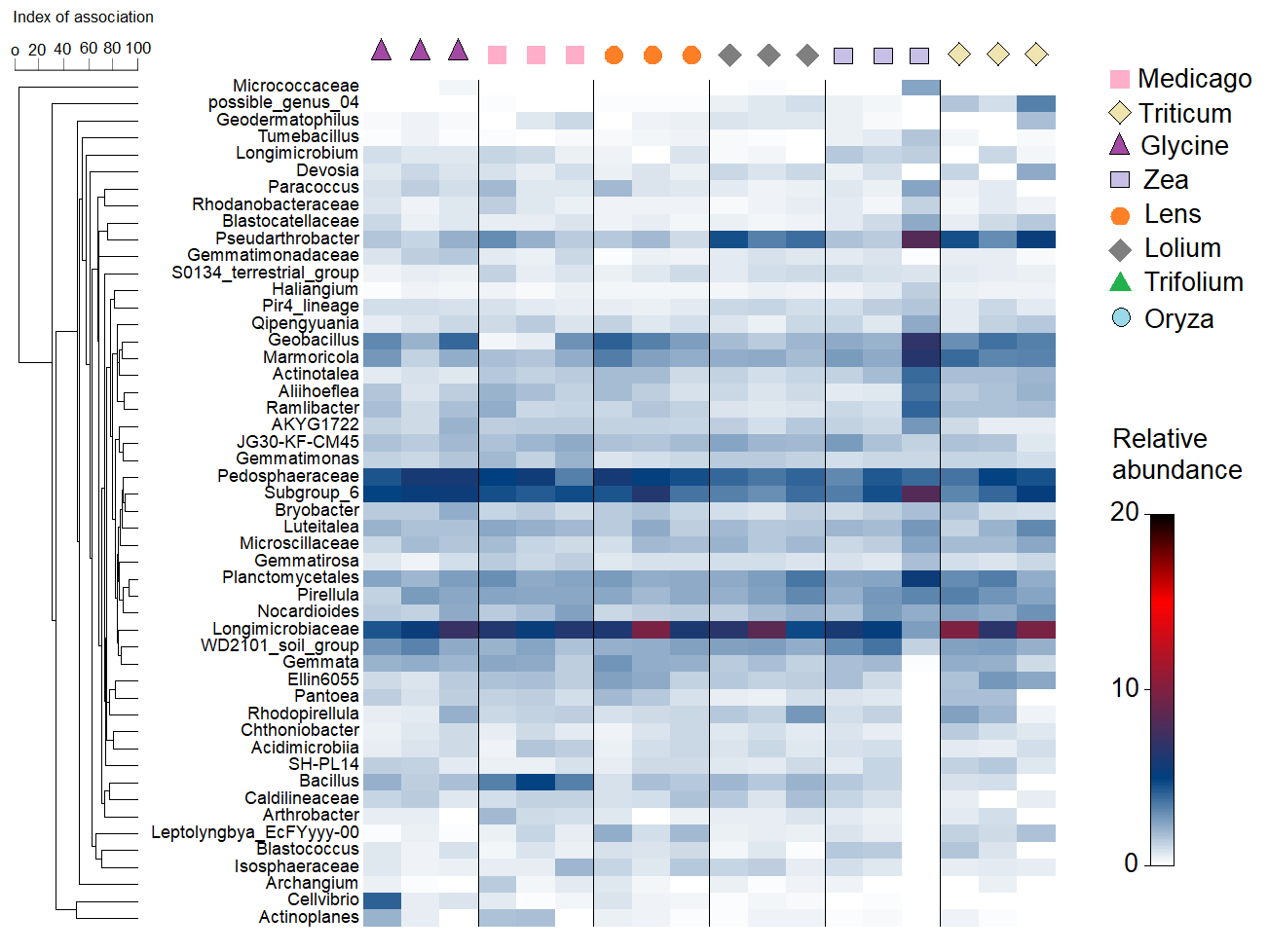
**

**Figure S4.** Heatmap showing relative abundance of the 50 most frequent Amplicon Sequence Variants (ASVs) in the bacterial community in each of the eight soil histories. The hierarchical grouping of variables is based on Whittaker’s association index.

**Table S1.** Topological parameters for the microbial networks in the conditioned soils.

| Parameters | Medicago | Triticum | Glycine | Zea | Lens | Lolium |
| --- | --- | --- | --- | --- | --- | --- |
| Nodes | 77 | 89 | 84 | 85 | 89 | 91 |
| Edges | 1022 | 1395 | 1141 | 1632 | 1809 | 1555 |
| Positive correlations | 70.16 | 70.11 | 69.76 | 57.6 | 75.57 | 78.71 |
| Negative correlations | 29.84 | 29.89 | 30.24 | 42.4 | 24.43 | 21.29 |
| Network diameter ^a^ | 5 | 7 | 6 | 6 | 5 | 6 |
| Characteristic path length ^b^ | 2.231 | 2.012 | 2.002 | 1.723 | 1.669 | 2.067 |
| Clustering coefficient ^c^ | 0.431 | 0.396 | 0.394 | 0.403 | 0.419 | 0.408 |
| Network density ^d^ | 0.175 | 0.178 | 0.164 | 0.229 | 0.231 | 0.19 |
| Modularity ^e^ | 0.997 | 0.786 | 0.9 | 1.537 | 0.339 | 0.452 |

^a^ The longest graph distance between any two nodes in the network (i.e. how far apart are the two most distant nodes)
^b^ Average number of edges in the shortest paths between all node pairs.
^c^ Degree of nodes tending to cluster together.
^d^ Measurement indicating the percentage of potential connections that actually exist among nodes in a network.
^e^ Degree of nodes tending to differentiate into different communities.
